# Multigrid simulations of non-Newtonian fluid flow and heat transfer in a ventilated square cavity with mixed convection and baffles

**DOI:** 10.1038/s41598-024-57322-5

**Published:** 2024-03-20

**Authors:** Nusrat Rehman, Rashid Mahmood, Afraz Hussain Majeed, Ilyas Khan, Abdullah Mohamed

**Affiliations:** 1 Department of Mathematics, Air University, PAF Complex E-9, Islamabad, 44000 Pakistan; 2 Department of Mathematics, College of Science Al-Zulfi, Majmaah University, 11952 Al-Majmaah, Saudi Arabia; 3 Research Centre, Future University in Egypt, New Cairo, 11835 Egypt

**Keywords:** Heat transfer, Baffles, Vented square cavity, Non‐Newtonian fluid, Mixed convection, Power law, Mechanical engineering, Applied mathematics

## Abstract

The impact of baffles on a convective heat transfer of a non-Newtonian fluid is experimentally studied within a square cavity. The non-Newtonian fluid is pumped into the cavity through the inlet and subsequently departs from the cavity via the outlet. Given the inherent non-linearity of the model, a numerical technique has been selected as the method for obtaining the outcomes. Primarily, the governing equations within the two-dimensional domain have been discretized using the finite element method. For approximating velocity and pressure, we have employed the reliable ${\mathbb{P}}_{2}$–${\mathbb{P}}_{1}$ finite element pair, while for temperature, we have opted for the quadratic basis. To enhance convergence speed and accuracy, we employ the powerful multigrid approach. This study investigates how key parameters like Richardson number (Ri), Reynolds number (Re), and baffle gap ${{\text{b}}}_{{\text{g}}}$ influence heat transfer within a cavity comprising a non-Newtonian fluid. The baffle gap (${b}_{g}$) has been systematically altered within the range of 0.2–0.6, and for this research, three distinct power law indices have been selected namely: 0.5, 1.0, and 1.5. The primary outcomes of the investigation are illustrated through velocity profiles, streamlines, and isotherm visualizations. Furthermore, the study includes the computation of the ${Nu}_{avg}$(average Nusselt number) across a range of parameter values. As the Richardson number (Ri) increases, ${Nu}_{avg}$ also rises, indicating that an increase in Ri results in augmented average heat transfer. Making the space between the baffles wider makes heat flow more intense. This, in turn, heats up more fluid within the cavity.

## Introduction

Convective heat transfer in cavities has become a magnet for researchers, attracting widespread interest and attraction. It’s a captivating field of study that has captured the attention of the scientific community, standing out as an exclusive and highly sought-after research area. Mixed convection heat transfer in vented cavities has gained noteworthy attention in practical engineering problems due to its amenable simplicity. In recent times, heat transfer through the introduction of obstacles within cavities has also achieved notable interest. The phenomenon of fluid flow around a rotating cylinder within a ventilated enclosure is commonly observed in a variety of real-world applications. These include thermal system design, rotary heat exchangers, air conditioning systems, electric cooling mechanisms, oil well drilling operations, solar collectors, and food processing industries. In each of these scenarios, understanding the behavior of the fluid flow is crucial for optimizing performance and efficiency. By reviewing this phenomenon, engineers and designers can develop innovative solutions to encounter precise requirements and improve the overall functionality of these applications^1^. Optimizing heat transfer within a vented enclosure can be achieved through strategic placement of baffles. These baffles help to endorse better airflow and increase the efficiency of heat transfer within the system. By purposeful insertion of baffles, the flow shapes can be directed in a way that maximizes heat exchange, leading to enhanced overall performance. Bassam^2^ carried out a computational study to scrutinize the laminar natural convection occurring around a horizontal cylinder. This cylinder was fitted with one or more than one fin (baffles) on its external surface, characterized by low thermal conductivity. It was discovered that the efficient positioning of single or dual baffles has a substantial impact on the reduction of entropy formation and heat transfer. Abraham and Sparrow^3^ conducted a thorough experiment to examine heat transfer in an oven-shaped enclosure. They also looked into how having or not having vents in the oven affected things. It was discovered that as the heating period progressed, the frequency of the periodic temperature changes brought on by the oven control circuit decreased. Saha et al.^4^ performed a numerical investigation to explore transverse mixed convection within a ventilated enclosure. Their focus was on a uniformly heated bottom wall under a fixed heat flux. Based on the computational findings, the position of the inlet and outlet has a substantial impact on both the heat transfer along the heated cavity wall and the dispersion of temperatures within the flow fields. Joodi^5^ conducted an investigation using COMSOL Multiphysics techniques to study how the geometry of baffles in the flocculation basin affects turbulence behavior. It was found that changing the inlet water velocity can significantly impact the turbulence structure of the water carrying the particles. Radhakrishnan et al.^6^ investigated mixed convection from a heat-generating device in a vented cavity with or without a baffle configuration using numerical and experimental methods. The existence or presence of a baffle placed at the central of the bottom wall has been observed to effectively channel the flow toward the heater. This configuration led to an enhancement in the freezing rate of up to 50% compared to the scenario where no baffle was present. Nougbléga et al.^7^ systematically analyze mixed convection within a baffled vented chamber where a uniform heat flow emanates from the left vertical wall, aiming to provide a quantitative assessment. The findings demonstrate that the horizontal walls where the heated baffles are located allow for the simultaneous creation of heated and isolated zone in the baffled vented cavity. Mahmood et al.^8^ performed a computational analysis of heat transfer within a magnetized staggered cavity with wavy insulation baffles. They addressed the partial differential equations that describe the flow and heat transfer phenomena within the cavity. Results indicate that when the Rayleigh number is smaller, the flow strength is greater. Aun et al.^9^ undertook a comprehensive investigation into the heat transfer dynamics within an enclosures, especially studying the impact of a vertical heated block and baffles. Results indicate that the baffles spread the flow inside the cavity and produce several circulation cells, which improve heat transfer. In a vented square enclosure with vertical walls that are differentially heated and an elastic baffle connected to a circular rod, Hamzeh et al.^10^ concentrate on the transient mixed convection. According to the research findings, it was evident that the comparison of the average Nusselt number in cavities with flexible baffles, rigid baffles, and no baffles showed that the cavity with the flexible baffle had the highest value. Palaniappan et al.^11^ look at the outcomes of a computational analysis of convection in ventilation square chamber with parallel insulated baffles. It is discovered that the behavior of ventilation cavities depends on more factors than only the size and placement of the baffles. It also greatly depends on how the ventilation cavity is set up.

The investigation of non-Newtonian fluid flow in vented cavities opens up exciting opportunities for advancing heat transfer and tumbling friction. It’s a fascinating path of research that holds boundless potential for innovation in these areas. Kefayati^12^ conducted an investigation into mixed convection involving non-Newtonian nanofluids within a square cavity driven by lids on two sides. The findings indicate that as the Richardson number increases, the influence of nanoparticles becomes more pronounced and significant. Yassen and Ismael^13^ studied how power law fluid design interacts in a trapezoidal cavity connected to a rectangular channel. The analysis demonstrates a significant improvement in heat transfer with the suggested baffled channel. Yang and Du^14^ conducted a study on heat transfer in enclosures using non-Newtonian fluids or nanofluids. Their discoveries suggest that enhancing the precision of fluid simulations in cavities can be achieved by quantifying the complex behavior of non-Newtonian nanofluids using empirical viscosity model. Shahabadi et al.^15^ explored into the intricate realm of flow and heat transfer, with a particular emphasis on a non-Newtonian fluid characterized by a power law. They introduce a twist by incorporating a flexible fin within a cavity. Flow patterns in natural convection are heavily influenced by both non-Newtonian fluid behavior and fin flexibility, mainly above the fin. Ghurban et al.^16^ numerically investigate mixed convective heat transfer in an externally vented square cavity occupied with non-Newtonian fluid and equipped with a baffle. Conclusions specifies that the power law index plays a substantial role in determining how strong the convectional transfer is.

Nanofluids within cavities, combined with baffles or fins, are of great importance in current research. Mahmoudi et al.^17^ conducted a numerical investigation that explored the complex interplay between mixed convection flow patterns and temperature distributions within a square cavity equipped with vents. Their study also incorporated the use of an external nanofluid consisting of copper and water. According to the investigations, the thermal properties and the flow field are significantly influenced by Re, Ri, as well as the solid concentration. Bellahcena et al.^18^ took on the challenge of exploring the fascinating world of forced convection heat transfer and fluid flows in a baffled shell and tube heat exchanger (STHE), using water-based $A{l}_{2 }{0}_{3}$ nanofluids. The findings indicate that using nanofluids in conjunction with baffling techniques in STHEs can improve heat transfer rates while consuming less energy. Ali et al.^19^ performed a computational investigation to observe mixed convection flow within a horizontal channel. This channel was configured with alternating baffles and exposed to an external magnetic field. It was discovered that alternated baffles greatly impacted the flow circulation by creating vortices and serpentine streamlines. The process of natural convection was numerically investigated by Gumir et al.^20^ through the utilization of a two-dimensional enclosure, which features finite solid wavy walls and is occupied with a hybrid water nanofluid. Experimental data showed that the heat transfer rate progressively decreased with the introduction of additional undulations. Biswas et al.^21^ propose an powerful method to enhance mixed convection heat transfer within a corrugated channel by segregating the overall flow into a primary flow and an injected flow, particularly under the assisting flow configuration. The findings indicate consistently superior performance with injection, leading to a heat transfer enhancement ranging from 50 to 218%. The extent of enhancement is contingent on features such as injection, Richardson number, and Reynolds number. Biswas et al.^22^ investigate the thermal control of a heating element by dividing the heater into multiple identical segments and studying it under mixed convection conditions. The findings suggest that heat transfer from segmental heating consistently surpasses that of uniform heating, whether applied to the left or right side of the enclosure. Biswas et al.^23^ explore the heat transfer characteristics of a porous cavity heated at the bottom and subjected to top injection of Cu-water nanofluid. The investigation employs the Brinkman–Forchheimer–extended Darcy model (BFDM) and assumes laminar flow conditions. Chakravarty et al.^24^ explore the effectiveness of side-injected cold fluid in boosting heat extraction from a truncated conical, internally heated porous bed within a restricted volume. The results suggest a notable enhancement in the extraction of temperature from the heat-generating porous bed with the additional introduction of cold fluid into the enclosure.

Today's researchers have the potential to find precise results with only a few computations because to the progress of the multigrid approach in current years. In the initial few repetitions, single-grid methods quickly converge, but as the correct solution of the finite-difference equations approaches, the rate of convergence steadily declines. In fact, the precision typically reaches an asymptotic limit after which further improvement is exceedingly expensive^25^. Certainly! The multigrid method is a powerful technique used in computational fluid dynamics (CFD) to solve complex fluid flow problems. Researcher have extensively studied and described the application of this method in CFD. They have provided detailed insights into the implementation and benefits of the multigrid method in CFD^26^. Wesseling and Oosterlee^27^ provides a brief overview of the geometric multigrid method’s development in CFD, focusing over the past decade. They discuss its application in both compressible and incompressible flow problems, along with the conforming multigrid solutions. It’s a valuable resource for understanding the recent developments in this field. Zhang et al.^28^ developed a 2D geometric multigrid model designed for solving the Poisson equation, particularly when an interface is present on a grid that employs structured adaptive mesh refinement. Their work is all about efficiency tackling this equation using a multigrid approach. It’s an important contribution to the field of numerical methods for interface problems.

Mandal et al.^29^ unveils valuable insights into the interaction between heat, flow, and motile microorganisms within porous environments, with potential applications in bio-inspired engineering and microfluidic systems. It has been observed that effective control of both local and global transport system can be attained by modifying the pertinent flow parameters and adjusting the number of undulations. In applications involving low-power magneto-hydrodynamic (MHD) thermal systems, the use of aspiration of the working media (without fans or pumps) can greatly increase heat transfer. In their investigation of its efficacy in the absence of aspiration, Biswas et al.^30^ take into account magnetic fields in addition to a traditional thermal cavity including a protruding heater positioned in the center of the bottom. According to the data, both with and without a magnetic field, aspiration enhances heat transmission during natural convection. Chattopadhyay et al.^31^ demonstrated the potential of directional wall motion control for manipulating fluid flow and improving thermal performance in wavy enclosures. The findings revealed that the direction of wall motion and the number of undulations in the wavy bottom surface have the greatest influence (over 70%) on the behavior of mixed convective flow, compared to other factors such as fluid properties and temperature difference. This emphasizes that the orientation of wall movement can play a crucial part in governing the thermal performance within the enclosure.

Selimefendigil and Öztop^32^ used the finite volume method to study how a partially curved layer with tiny pores (porous layer) affects heat transfer and the creation of disorder (entropy) in a ventilated space filled with a fluid containing a mixture of nanoparticles (hybrid nanofluid). The study also considered the influence of an angled magnetic field on this system. They found that when the speed of the nanofluid increased, the area of the swirling flow region (recirculation zone) below the inlet and the whirlpool (vortex) near the top corner became larger. Chung and Choi^33^ investigated the airflow patterns induced by a retractable baffle in a large-scale, mechanically ventilated barn to assess its effectiveness in mitigating heat stress. Farhany et al.^34^ used numerical simulations to study how heat transfer and temperature distribution (conjugate heat transfer characteristics) behave in a tilted enclosure filled with a magnetic liquid (ferrofluid) and a porous material, subject to uneven heating. The study found that increasing the modified Rayleigh number, Darcy number, and fin length all led to a higher average Nusselt number. Conversely, increasing the Hartmann number resulted in a decrease in the average Nusselt number. Muhja and Farhany^35^ used numerical simulations to investigate how the angle of a slanted plate (baffle) inside a square enclosure affects the natural convection heat transfer of a nanofluid. The average Nusselt number rises with both increasing Rayleigh number and thermal conductivity ratio. This happens because a stronger driving force (Rayleigh number) and better heat conduction across the interface (thermal conductivity ratio) enhance heat transfer between the wall and the fluid.

In accordance with the material displayed above, it appears that there is not much research out there on using multigrid technique to investigate the non-Newtonian fluid flow in a cavity that is vented with different baffle gaps. It’s definitely an area that could use more investigation to understand how presence of baffles affects the flow behavior and how multigrid approach can be applied to analyze it. Hence, the goal of current research work is to computationally analyze the convection of non-Newtonian fluids in a vented cavity with baffles. By utilizing the multigrid approach, we aim to analyze the intricate flow characteristics and understand the impact of baffles on the fluid behavior. The dynamics of a viscous fluid within a ventilated cavity with heated baffles is examined. Specially looking at how different gap values between baffles affect the flow characteristics. The enclosure's left-wall vent serves as the fluid's entry point, with subsequent expulsion facilitated by a vent located at the top-right section. The following is how the paper is set up: “Introduction” section of the paper presents an innovative twist by reviewing the motivational literature. “Problem description” section tackle the problem description. “Mathematical description” section provide a clear mathematical formation for the problem. In “Numerical procedure and convergence test” section, we introduce a set of numerical techniques that pave the way for accurate and efficient simulations. “Results and discussion” section is unveiling the amazing outcomes of this study. In the final section, we draw compelling conclusion that ties together all our findings.

## Problem description

**Figure 1:**
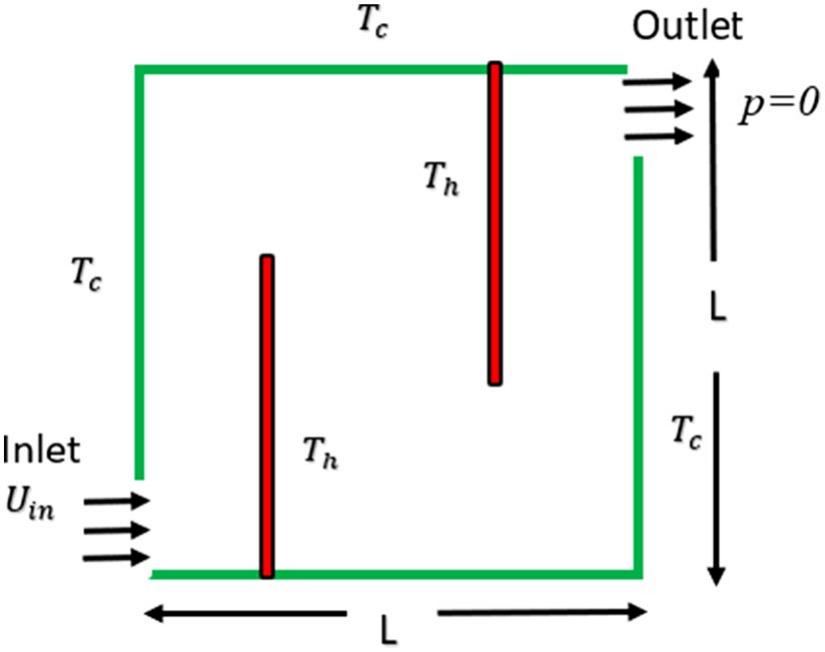
Physical structure of the problem.

Figure 1 displays the ventilated square cavity, depicted within the framework of the cartesian coordinate system. The square cavity has a length indicated by the variable $L$. The cavity contains non-Newtonian fluid, which enters through the inlet located on the bottom left-side wall and exits through the designated area on the right wall. In this scenario, all the walls have a temperature of zero, meanwhile, the baffles are maintained at an elevated temperature of $T$ = 1. In this research, the baffles have a non-dimensional width of 0.02 and a length of 0.75. For this study, three distinct baffle gaps were used.

When the baffle gap is set to 0.2, the left and right baffle’s center is at $x$ = 0.38 and 0.58 respectively. When the baffle gap is set to 0.4, the left and right baffle’s center is at $x$ = 0.28 and 0.68 respectively. When the baffle gap is set to 0.6, the left and right baffle’s center is at $x$ = 0.18 and 0.78 respectively.

## Mathematical description

The fundamental equations governing the behavior of non-Newtonian fluids encompass the law of conservation of mass (continuity), momentum, and energy equations^36^ in dimensional form is given below:

1 $$\frac{\partial u}{\partial x}+\frac{\partial v}{\partial y}=0$$

2 $$\rho \left[u\frac{\partial u}{\partial x}+v\frac{\partial u}{\partial y}\right]+\frac{\partial p}{\partial x}=\left(\frac{\partial {\tau }_{xx}}{\partial x}+\frac{\partial {\tau }_{xy}}{\partial y}\right)$$

3 $$\rho \left[u\frac{\partial v}{\partial x}+v\frac{\partial v}{\partial y}\right]+\frac{\partial p}{\partial x}=\left(\frac{\partial {\tau }_{xy}}{\partial x}+\frac{\partial {\tau }_{yy}}{\partial y}\right)+\rho \beta g(T-{T}_{C})$$

4 $$\rho {c}_{p}\left[u\frac{\partial T}{\partial x}+v\frac{\partial T}{\partial y}\right]=k\left[\frac{{\partial }^{2}T}{\partial {x}^{2}}+\frac{{\partial }^{2}T}{\partial {y}^{2}}\right]$$

where ${\tau }_{ij}$ is the stress component, ${\tau }_{ij}=2\eta {\varepsilon }_{ij}$ and ${\varepsilon }_{ij}$ is the deformation tensor given as ${\varepsilon }_{ij}=\left(\frac{\partial {u}_{i}}{\partial xj}+\frac{\partial {u}_{j}}{\partial xi}\right)$ ${c}_{p}$ is the specific heat, while $\beta$ and $k$ represent thermal expansion coefficient and thermal conductivity, respectively. Density is represented by $\rho .$ Pressure is represented by $p$ and $u$, $v$ represents the $x$ and $y$ components of velocity respectively.

To make Eqs. (1)-(4) dimensionless, we can scale the variables as follows:

5 $$ \overline{X} = \frac{x}{L},\quad \overline{Y} = \frac{y}{L},\quad \overline{U} = \frac{u}{{U_{in} }},\quad \overline{V} = \frac{v}{{U_{in} }},\quad \overline{P} = \frac{p}{{\rho U_{in}^{2} }},\quad \theta = \frac{{T - T_{C} }}{{T_{H} - T_{C} }} $$

In this context, the dimensionless variables $\overline{X }$, $\overline{Y }, \overline{U }, \overline{V }, \overline{P }$ and θ represent the coordinates, $x$ velocity component, $y$ velocity component, pressure and normalized temperature, respectively.

The equations in dimensionless form are as follows:

6 $$\frac{\partial \overline{U} }{\partial \overline{X} }+\frac{\partial \overline{V} }{\partial \overline{Y} }=0$$

7 $$\left[\overline{U }\frac{\partial \overline{U} }{\partial \overline{X} }+V\frac{\partial \overline{U} }{\partial \overline{Y} }\right]+\frac{\partial \overline{P} }{\partial \overline{X} }=\frac{1}{Re}\left(2\frac{\partial }{\partial \overline{X} }\left(\frac{\eta }{m}\frac{\partial \overline{U} }{\partial \overline{X} }\right)+\frac{\partial }{\partial \overline{Y} }\left(\frac{\eta }{m}\left(\frac{\partial \overline{U} }{\partial \overline{Y} }+\frac{\partial \overline{V} }{\partial \overline{X} }\right)\right)\right)$$

8 $$\left[\overline{U }\frac{\partial \overline{V} }{\partial \overline{X} }+\overline{V }\frac{\partial \overline{V} }{\partial \overline{Y} }\right]+\frac{\partial \overline{P} }{\partial \overline{Y} }=\frac{1}{Re}\left(2\frac{\partial }{\partial \overline{Y} }\left(\frac{\eta }{m}\frac{\partial \overline{V} }{\partial \overline{Y} }\right)+\frac{\partial }{\partial \overline{X} }\left(\frac{\eta }{m}\left(\frac{\partial \overline{U} }{\partial \overline{Y} }+\frac{\partial \overline{V} }{\partial \overline{X} }\right)\right)\right)+Ri\times \theta $$

9 $$\left[\overline{U}\frac{\partial \theta  }{\partial \overline{X} }+\overline{V}\frac{\partial \theta  }{\partial \overline{Y} }\right]=\frac{1}{Pr\times Re}\left[\frac{{\partial }^{2}\theta }{\partial {\overline{X} }^{2}}+\frac{{\partial }^{2}\theta }{\partial {\overline{Y} }^{2}}\right]$$

where $\eta$ represents the apparent viscosity given as, $\eta =m{\left\{2\left[{\left(\frac{\partial \overline{U} }{\partial \overline{X} }\right)}^{2}+{\left(\frac{\partial \overline{V} }{\partial \overline{Y} }\right)}^{2}\right]+{(\left(\frac{\partial \overline{U} }{\partial \overline{Y} }\right)+\left(\frac{\partial \overline{V} }{\partial \overline{X} }\right))}^{2}\right\}}^{\frac{(n-1)}{2}}$, $m$ represents the constancy parameters and $n$ represents the power-law index. Table 1 provides a condensed overview of the non-dimensionalized boundary conditions for the current problem.

**Table 1:** According to the problem specification, the dimensionless boundary condition is as follows:

Boundary	Flow conditions	Thermal conditions
Top wall	$0 \le \overline{X} \le 1,\quad \overline{Y} = 1,\quad \overline{U} = \overline{V} = 0$	$\theta = 0$
Bottom wall	$0 \le \overline{X} \le 1,\quad \overline{Y} = 0,\quad \overline{U} = \overline{V} = 0$	$\theta = 0$
Left wall	$\overline{X} = 0,\quad 0.1 \le \overline{Y} \le 1,\quad \overline{U} = \overline{V} = 0,$	$\theta = 0$
Right wall	$\overline{X} = 1,\quad 0 \le \overline{Y} \le 0.9,\quad \overline{U} = \overline{V} = 0,$	$\theta = 0$
Inlet	$\overline{U}_{in} = 1,\quad \overline{X} = 0,\quad 0 \le \overline{Y} \le 0.1,\quad \overline{U} = \overline{V} = 0$	$\theta = 0$
Outlet	$\overline{X} = 1, 0.9 \le \overline{Y} \le 1 ,\overline{P} = 0,$ $\frac{{\partial \overline{U}}}{{\partial \overline{X}}}$ = 0, $\frac{{\partial \overline{U}}}{{\partial \overline{Y}}} = 0$	$\frac{\partial \theta }{{\partial \overline{X}}} = 0$
Baffles	$\overline{U} = \overline{V} = 0$	$\theta =1$

The non-dimensional quantities that arise in the governing equation are Reynold, Richardson, Grashof and Prandtl number. These numbers are described below:

$$Re= \frac{\rho {L}^{n}{\overline{U} }_{in}^{2-n}}{m}$$, $$Gr=g\beta \left({T}_{H}-{T}_{C}\right){L}^{3} {\left[\frac{\rho }{m}{\left(\frac{{\overline{U} }_{in}}{L}\right)}^{1-n}\right]}^{2}$$

$$Ri=\frac{g\beta \left({T}_{H}-{T}_{C}\right)L}{{\overline{U} }_{in}^{2}}=\frac{Gr}{{Re}^{2}}$$, $$Pr=\frac{{C}_{P}m}{k}{\left(\frac{{\overline{U} }_{in}}{L}\right)}^{n-1}$$

## Numerical procedure and convergence test

**Figure 2:**
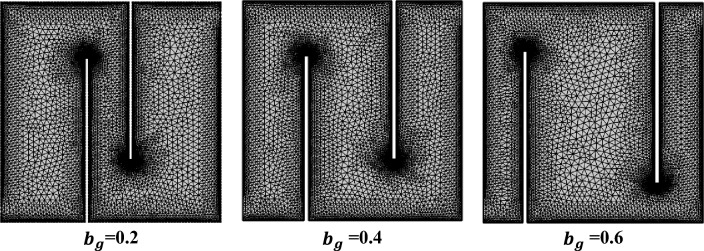
Computational mesh at Coarse level.

To solve** c**omplex fluid flow problems, the Galerkin’s finite element method converts the continuous partial differential equations for velocity and pressure into a set of linear algebraic equations that can be competently solved through computational methods. Velocity is discretized using ${\mathbb{P}}_{2}$ element, while pressure is approximated with the ${\mathbb{P}}_{1}$ element. The temperature approximations are also chosen to be quadratic for better accuracy of the thermal field. The computational mesh at the extra fine level can be seen in Fig. 2. The problem's overall degree of freedom at each of its refinement level is shown in Table 3. Selecting a single element from the computational mesh and subsequently subdividing each element into four parts by connecting the midpoints of opposing edges, the grid refinement process is continued.

Newton's method with appropriate damping is used to iteratively crack the underlying system of nonlinear algebraic equations. The linearized equations at each iteration are solved using PARDISO^37–41^, a direct solver that can efficiently solve large, sparse systems of equations. The whole implementation is employed in COMSOL Multiphysics Solver 5.6.

To validate the model and code, we compared simulated data with published results, particularly the average Nusselt number from Majeed et al.^39^. Table 2 demonstrates close correspondence between simulated and literature-reported average Nusselt numbers (${Nu}_{avg}$) at various Reynolds numbers (Re). This high level of agreement confirms the robustness and reliability of the present model and numerical scheme for achieving accurate solutions in this context.

**Table 2:** Analyzing the impact of parameter variations on Nusselt number at Pr = 5, Ri = 1.

Re	Majeed et al.^39^	Present results
5	11.6278655	11.6278758
10	15.7421133	15.7421200
15	18.7397606	18.7397812
20	21.1463851	21.1464263
25	23.2662513	23.2663275

Table 3 provides an overview of the total elements and degrees of freedom at each refining level. It is evident that as the refining level increases, the count of degrees of freedom and elements also increases. This is particularly notable when considering the expansion of the baffle’s gap.

**Table 3:** Degrees of freedom (DOF) and element count (EL).

Refinement levels	${{\varvec{b}}}_{{\varvec{g}}}=0.2$		${{\varvec{b}}}_{{\varvec{g}}}=0.4$		${{\varvec{b}}}_{{\varvec{g}}}=0.6$	
	#EL	#DOF	#EL	#DOF	#EL	#DOF
Extremely coarse	588	3539	612	3848	616	3733
Extra coarse	790	4747	784	4699	804	4862
Coarser	1162	6821	1150	6743	1162	6875
Coarse	2032	11,637	2030	11,618	2000	11,549
Normal	2904	16,400	2994	16,840	2882	16,353
Fine	4428	24,513	4,486	24,776	4426	24,611
Finer	11,890	64,427	12,068	65,299	11,944	64,859
Extra finer	31,240	165,791	31,596	167,571	31,240	166,151
Extremely fine	38,060	199,891	38,112	200,153	38,342	201,661

The results demonstrate a robust agreement, supporting the conclusion that the present model and numerical approach are dependable for obtaining solutions to the current problem with a suitable level of accuracy. Table 4 presents the assessment of grid resolution for kinetic energy for ${b}_{g}$ = 0.2, with Prandtl number (Pr) equal to 5, Reynolds number (Re) at 50, and Richardson number (Ri) set at 1. This configuration ensures that the deviation of selected quantities between grid levels 8 and 9 is negligibly small. Consequently, simulations are executed at grid level 8 to optimize computational resources. The table includes the corresponding number of elements (#EL) and total unknowns or degrees of freedom (#DOFs).

**Table 4:** Grid convergence test for the modeled problem for ${b}_{g}$ = 0.2$. {\text{Pr}}=5, Ri=1$ and Re = 50.

Levels	No. of elements	Degrees of freedom	Kinetic energy
1	588	3539	0.381081
2	790	4747	0.379966
3	1162	6821	0.378316
4	2032	11,637	0.378146
5	2904	16,400	0.377026
6	4428	24,513	0.375676
7	11,890	64,427	0.374562
8	31,240	165,791	0.373929
9	38,060	199,891	0.373888

### Nonlinear solver

The possibilities for solving Navier–Stokes equations using outer non-linear solvers are rather constrained. The above includes both the Fixed-Point iteration and the Newton iteration. Both of them are incredibly popular and are regularly used in numerical calculations. Since the Newton iteration achieves convergence more quickly than the fixed-point iteration, it is frequently chosen.

The one step Newton’s execution is as follows:

1. Given an initial estimate ${X}^{\left(0\right)}$, set $k=0$
2. Address the linear subproblem: $${\mathcal{J}}(X^{\left( k \right)} )$$$$\Delta X^{\left( k \right)} = - {\mathcal{F}}(X^{\left( k \right)}$$).
3. Update the iterate to achieve: $X^{k + 1} = X^{k} + \sigma \Delta X^{\left( k \right)}$
4. Set *k* = *k* + *1*, go to step 2.

This process should continue until the norm of $\Vert {X}^{k+1}-{X}^{k}\Vert$ is below a certain threshold. Here $\sigma$ is a damping parameter and $\mathcal{J}$ is Jacobean matrix of the non-linear system.

### Multigrid

**Figure 3:**
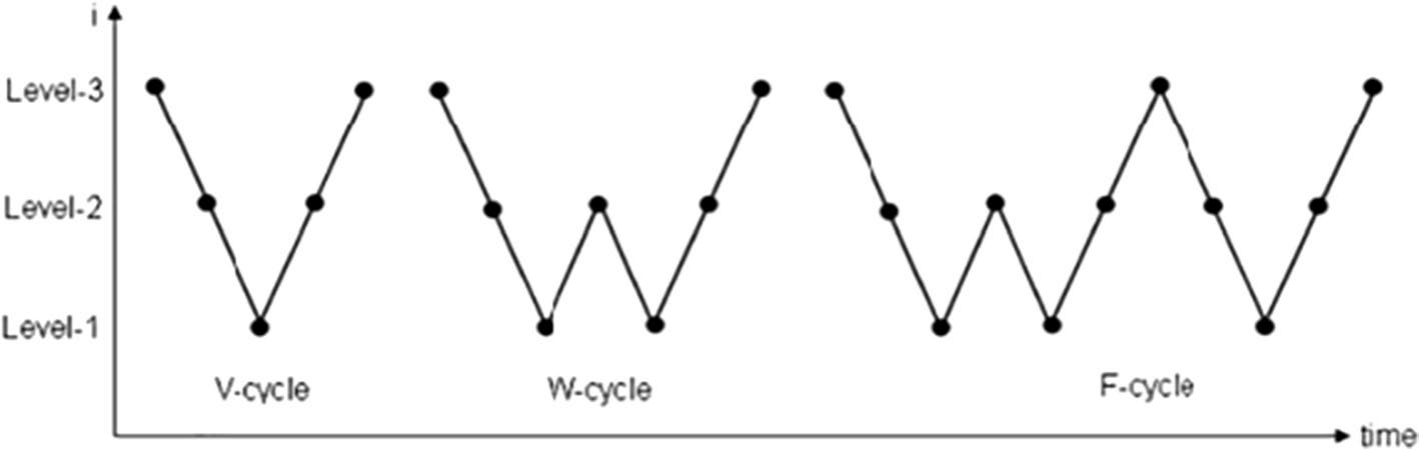
A diagram showing the steps involved in the three main types of multigrid cycles: the V-cycle, the W-cycle, and the F-cycle.

Multigrid is a super-efficient solver for PDEs with a efficient convergence rate, regardless of problem size. The multigrid approach works by creating a hierarchy of grid through regular refinement of the coarse mesh. After applying smoothing on different mesh levels, a restriction on the residual is imposed. The solution is found on the coarsest grid using a direct sparse linear solver if the degree of freedom count is sufficiently low. After post-smoothing, the prolongation step is carried out, which results in a improved approximation. The procedure keeps going till the entire multigrid iteration cycle (V, W, or F) is completed. An outline of the initial iteration of the multigrid algorithm's implementation is shown in Fig. 3. The multigrid method is widely recognized as one of the quickest linear solvers for CFD problems^27^. It’s pretty impressive. Regarding multigrid approaches, more information is provided by Hackbush^42,43^. To address the fluid flow problem, which is described in the subsequent sections, we used the multigrid technique. For pre-smoother and post-smoother, the most famous SOR (successive over relaxation) method were used.

Let’s give some consideration to the linear system

10 $${A}_{j}{{\varvec{U}}}_{j}= bj$$

- Set a starting value or guess ${{\varvec{U}}}_{j}^{o}$ on fine grid level $j$ and perform pre-smoothing to enhance the accuracy of the primary approximation
  11 $$ {\varvec{U}}_{j}^{k + 1} = S_{j} \left\{ {{\varvec{U}}_{j}^{k} } \right\},\quad k = 0, \ldots ,m - 1 $$
  where ${S}_{j}$ is the smoothing operator.
- Pre-smoothing adequately smooths the very high frequency of the residual such that the error's high frequency may be seen on the coarse grid.
  12 $$ r_{j - 1} = R_{j}^{j - 1} \left\{ {b_{j} - A_{j} {\varvec{U}}_{j}^{m} } \right\}, $$

The restriction operator ${R}_{j}^{j-1}$, converts a finer grid into a coarser grid and provides an approximate value.

- Solve the system to obtain the correction u*_j−1_ on the coarser grid correction.
  13 $$ {\text{A}}_{{{\text{j}} - {1}}} {\mathbf{u}}*_{{{\text{j}} - {1}}} = {\text{r}}_{{{\text{j}} - {1},}} $$
- Prolongate the correction u*_j−1_ to the finer grid
  14 $$ {\mathbf{U}}_{{\text{j}}}^{{{\text{m}} + {1}}} = {\mathbf{u}}^{{\text{m}}}_{{\text{k}}} + \alpha_{{\text{k}}} {\text{P}}^{{\text{j}}}_{{{\text{j}} - {1}}} {\mathbf{u}}*{\text{j}} - {1}, $$
  where P^j^_j−1_ denotes the prolongation operator used to interpolate values from a coarser grid to the next finer grid. and α_j_ is the damping parameter.
- Get the final solution **u**_j_^m+1+n^ by executing the post-smoothing steps

These steps are iteratively applied across various grid levels to expedite the reduction of errors.

The fundamental concept behind the development of the multigrid V-cycle (MGV) and full multigrid (MGF) algorithms is as follows:

**Algorithm 1:**
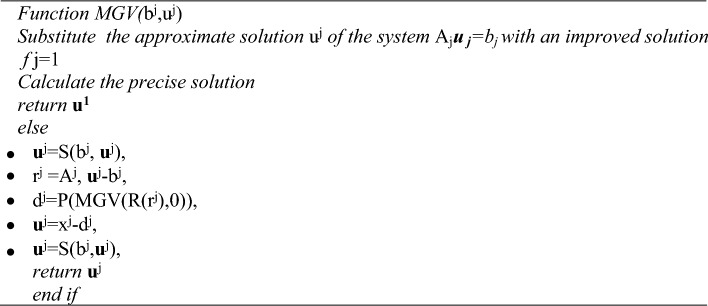
Multigrid V-Cycle

**Algorithm 2:**
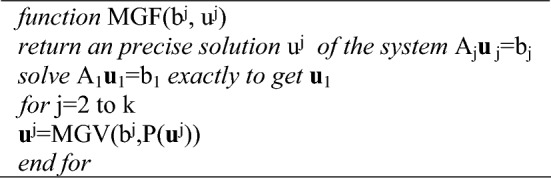
Full Multigrid (FMG)

## Results and discussion

In this research, the main object is to explore the fluid flow and convective heat transfer characteristics of a vented square cavity equipped with baffles of different sizes. The fluid being considered is non-Newtonian, and the flow is laminar and steady. The fluid enters through the bottom of the left wall and exists through the top of the right wall. This section provides a graphical representation of the results, including velocity profiles, streamline, isotherms and line graphs. This section also provides a comprehensive analysis of how various parameters influence the Nusselt number.

The power law index, $n$, is a dimensionless number that describes how the shear stress of a fluid changes with the shear rate. A Newtonian fluid has a power law index of 1, which means that the shear stress is directly proportional to the shear rate. A shear-thinning fluid has a power law index of less than 1, which means that the shear stress decreases as the shear rate increases. A shear-thickening fluid has a power law index of greater than 1, which means that the shear stress increases as the shear rate increases. Table 5 represents the number of iterations taken by the non-linear solver for different power law indices and three different gaps between the baffles. It is observed that for the shear thinning case (*n* < 1), more iterations are required for convergence, while for the shear thickening case (*n* > 1), fewer iterations are required for convergence.

**Table 5:** Number of iterations for nonlinear solver (Newton’s Method).

Power law index ($$n)$$	$${{\varvec{b}}}_{{\varvec{g}}}=0.2$$	$${{\varvec{b}}}_{{\varvec{g}}}=0.4$$	$${{\varvec{b}}}_{{\varvec{g}}}=0.6$$
0.5	31	29	28
1	12	11	12
1.5	11	11	11

**Figure 4:**
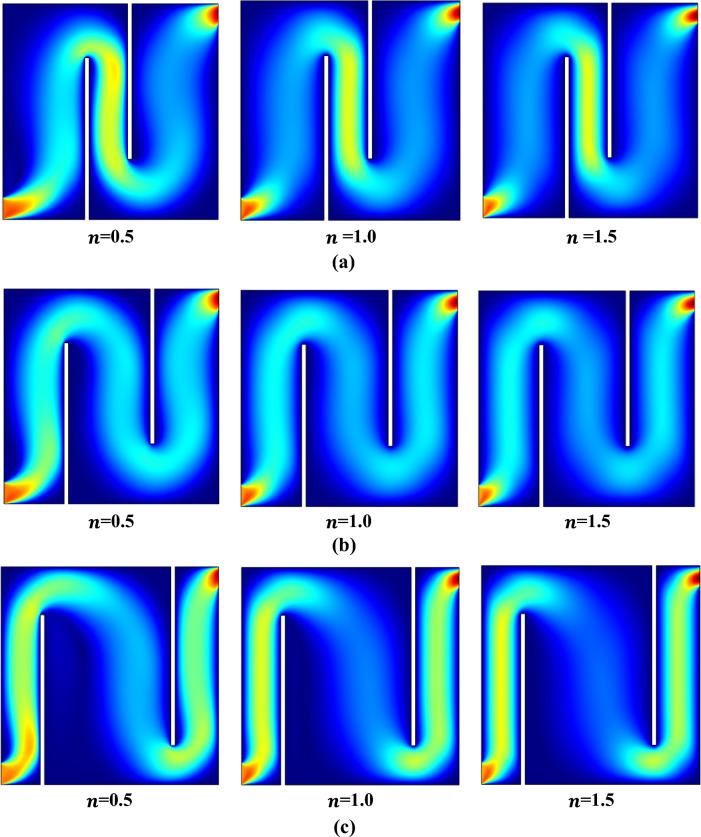
(**a**) Velocity profiles with baffle gap 0.2, Ri = 1 and Pr = 5.0. (**b**) Velocity profiles with baffle gap 0.4, Ri = 1 and Pr = 5.0. (**c**) Velocity profiles with baffle gap 0.6, Ri = 1 and Pr = 5.0.

In Fig. 4a–c, illustrates the Influence of various baffle gap sizes and power law indices on the velocity profile can be observed with fixed Prandtl number 5 and Richardson number Ri = 1. The power law indices are increasing from left to right ($n$=0.5, 1 and 1.5) and vertically, there are three increasing baffle gap sizes, which are: ${b}_{g}=0.2$, 0.4 and 0.6. By expanding the baffle gap, in the presence of baffles, a reduction in boundary layer thickness within velocity profiles is observed, leading to a shift in velocity patterns that may impact heat transfer or mixing efficiency.

**Figure 5:**
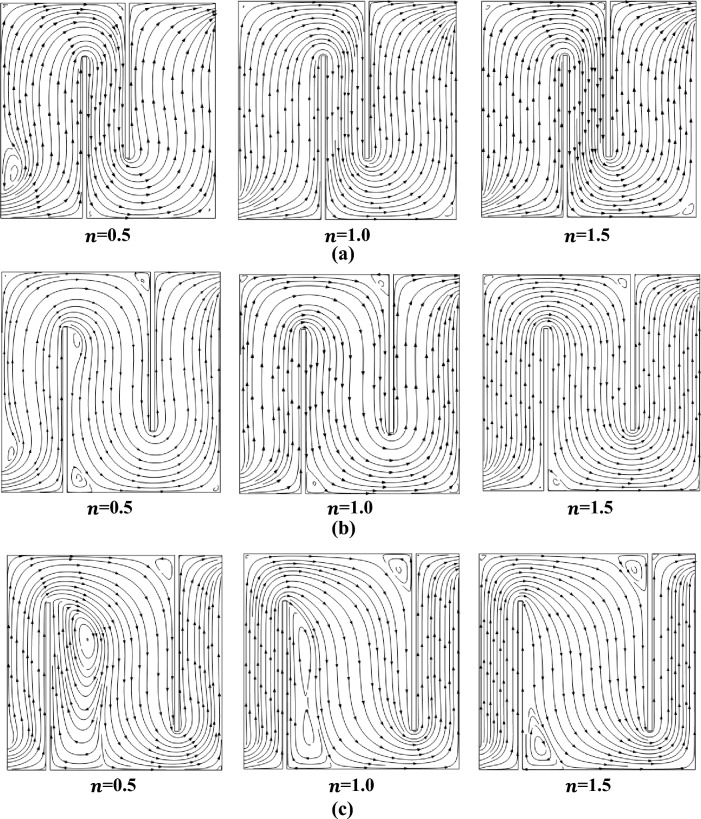
(**a**) Influence on Contours with baffle gap 0.2, Ri = 1, and Pr = 5.0. (**b**) Influence on Contours with baffle gap 0.4, Ri = 1, and Pr = 5.0. (**c**) Influence on Contours with baffle gap 0.6, Ri = 1 and Pr = 5.0.

In Fig. 5a–c, the impact of different baffle gaps and power law indices on the streamlines profile can be observed with fixed Prandtl number 5 and Richardson number *Ri* = 1. The power law indices vary in an ascending order from left to right, with values of $n$ equal to 0.5, 1, and 1.5. Progressing from the uppermost to the lowermost, there are three increasing baffling gaps denoted as ${b}_{g}$, with values of 0.2, 0.4, and 0.6. Vortices may be seen forming at the top, bottom, and all around the baffles. As we increase the baffle size, clockwise and anticlockwise vortices appear around the cavity. The best-case situation occurs at a baffle gap of 0.6, when larger and more vortices are produced as the power-law index rises.

**Figure 6:**
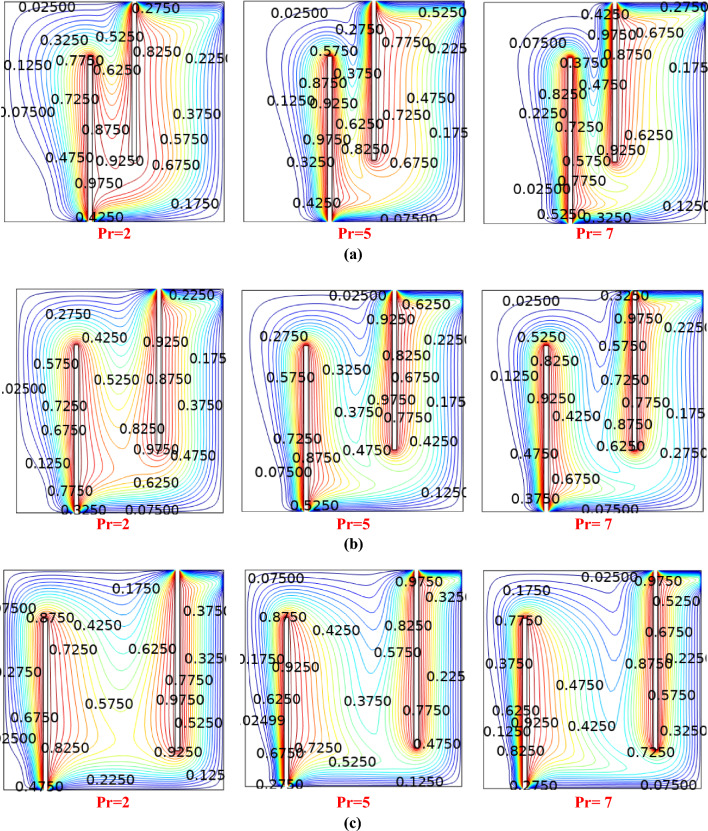
(**a**) Influence on Contours with baffle gap 0.2 and Ri = 1. (**b**) Influence on Contours with baffle gap 0.4 and Ri = 1. (**c**) Influence on Contours with baffle gap 0.6 and Ri = 1.

In Fig. 6a–c, the impact of varied baffle gaps and Prandtl number on the isotherms can be observed. The results are calculated for shear thickening *(n* > *1*) with *Ri* = *1*. In the vertical direction, there are three consecutive increments in the baffling gap sizes, specifically marked as ${b}_{g}$, with values of 0.2, 0.4, and 0.6 from top to bottom. Increased the Prandtl number can lead to reduction in the thermal boundary layer thickness, resulting in a denser distribution of isothermal lines in the vicinity of the heat source. At higher Prandtl numbers, the isothermal lines vanish from the lower region of the cavity. This phenomenon occurs because the highly viscous fluid confines the thermal boundary layer to a compact area.

**Figure 7:**
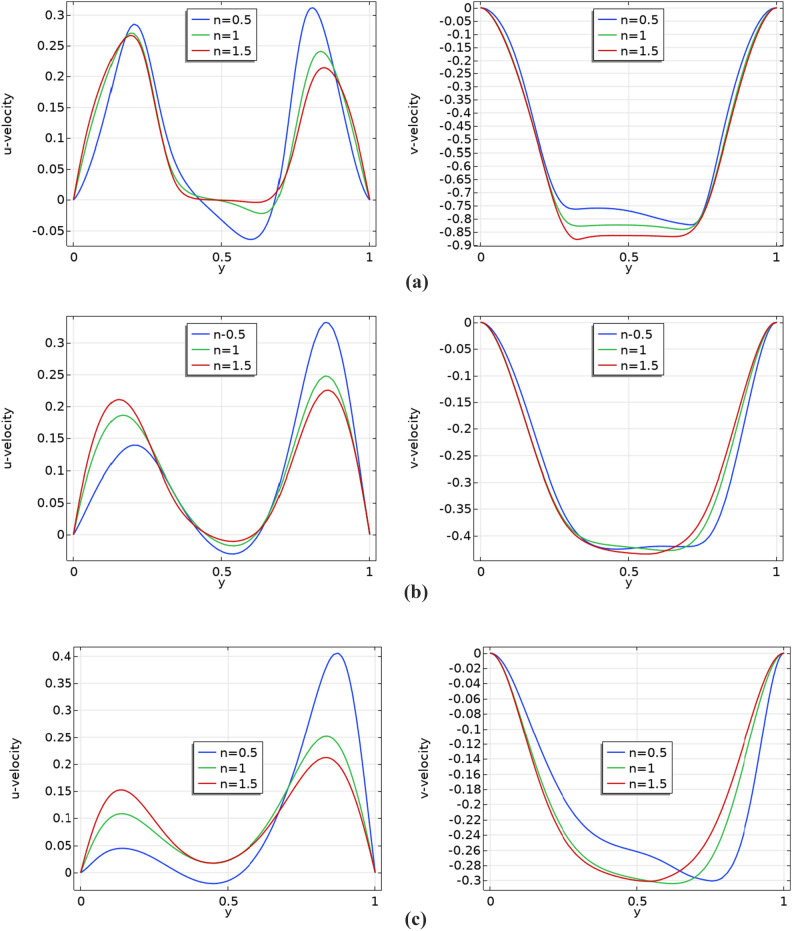
(**a**) Line graphs along $Y$-Axis at $x$ = 0.5 for a Baffle Gap of 0.2: $u$ and $v$ components. (**b**) Line graphs along $Y$-Axis at $x$ = 0.5 for a Baffle Gap of 0.4: $u$ and $v$ components. (**c**) Line graphs along $Y$-Axis at $x$ = 0.5 for a Baffle Gap of 0.6: $u$ and $v$ components.

Many researchers find it incredibly captivating to observe the velocity behavior along a straight line within 2D domain. It’s a truly intriguing phenomenon that has piqued the interest of numerous scholars. When considering a straight line, it can be oriented either horizontally or vertically. The attachment of these baffles to the horizontal wall significantly affects the velocity profile when plotting a horizontal line graph. Therefore, velocity measurements were taken along the vertical cross-section at a position where x equals 0.5. Figure 7a–c depict the $u$ and $v$ velocity components along the $y$-axis at $x$ = 0.5 for baffle gap sizes of ${b}_{g}$ = 0.2, 0.4, and 0.6. Each baffle gap exhibits three distinct curves, corresponding to power law indices of $n$ = 0.5, 1.0, and 1.5. The curves behave differently after the 0.5 demarcation.

**Figure 8:**
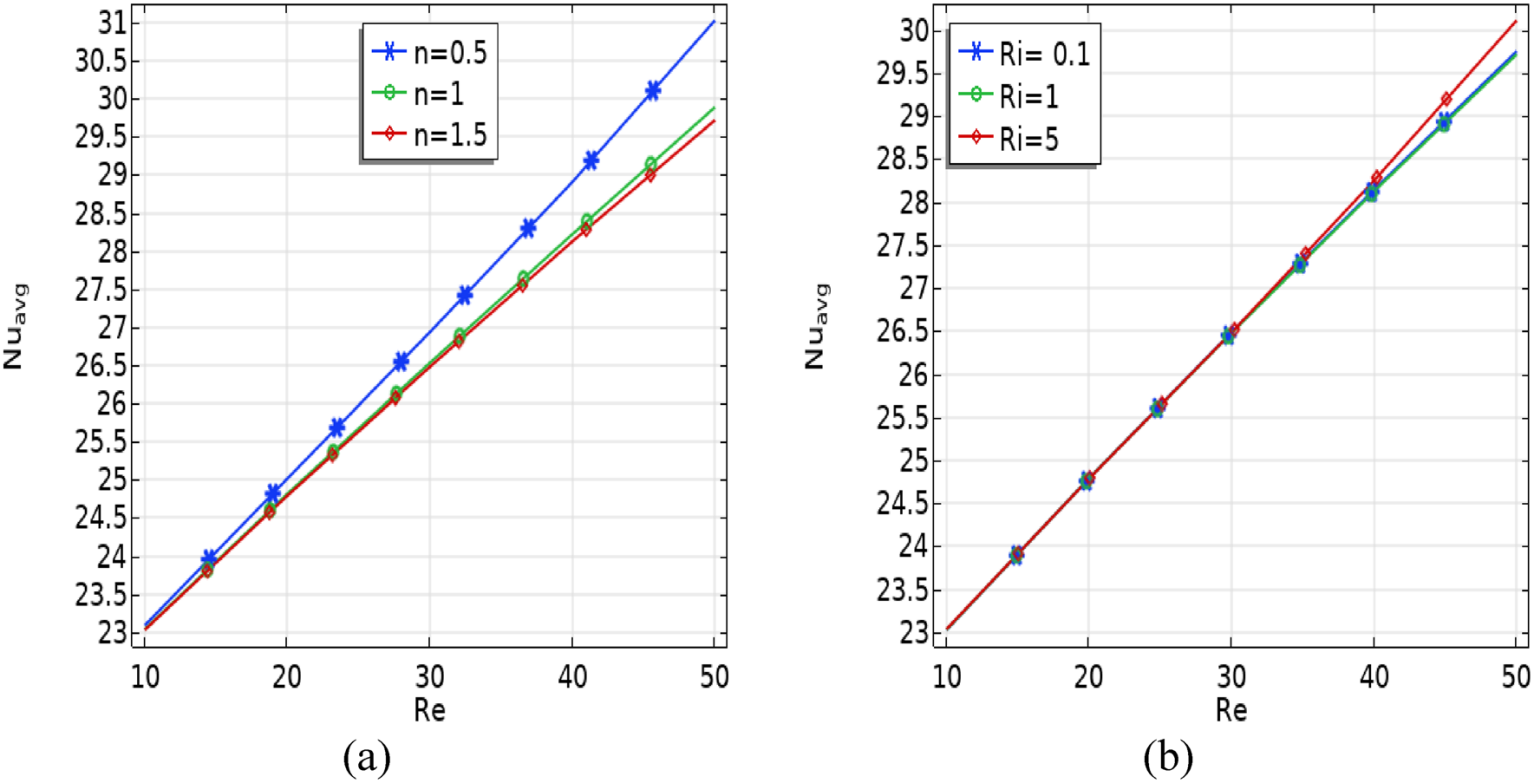
Average Nusselt number versus Reynolds number (**a**) for different power law indices $n$ **(b)** for different Richardson number (Ri).

In Fig. 8a, the influence of Reynolds number (Re) on the average Nusselt number at the baffles is illustrated for various power law indices ($n$). Findings indicate that expanding the Reynolds number (Re) leads to an enhancement in the Nusselt number value for the left wall. Likewise, improving the value of power law indices ($n$) ensures better performance of heat transfer. Figure 8(b) displays how the Re (Reynolds number) affects the average Nusselt number (${Nu}_{avg}$) at the baffles across different Richardson number values. Notably, the curves exhibit a clear linear correlation, as the graph consistently rises with increasing Ri.

**Figure 9:**
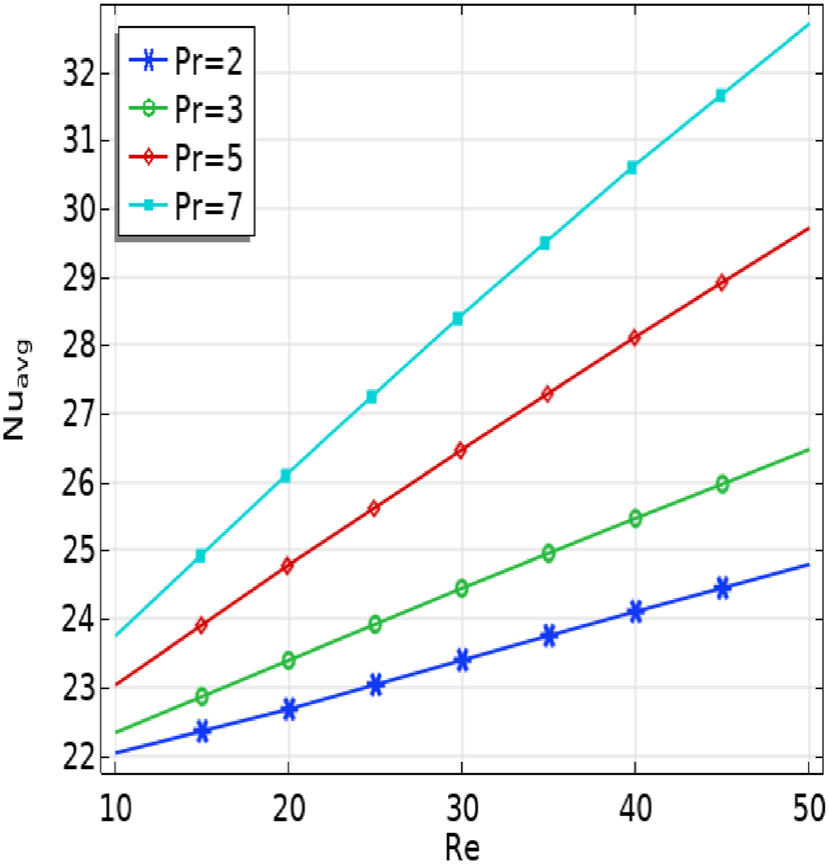
The effect of Prandtl (Pr) number and Ri on ${Nu}_{avg}$ number.

Figure 9 illustrates how the average Nusselt number at the baffles varies with different Prandtl number ($2\le Pr\le 7)$ values as influenced by the Reynolds number (Re). The results demonstrate that higher Reynolds numbers (Re) result in an increased Nusselt number. Additionally, it is noticeable that the curves rise as the Prandtl number increases.

**Figure 10:**
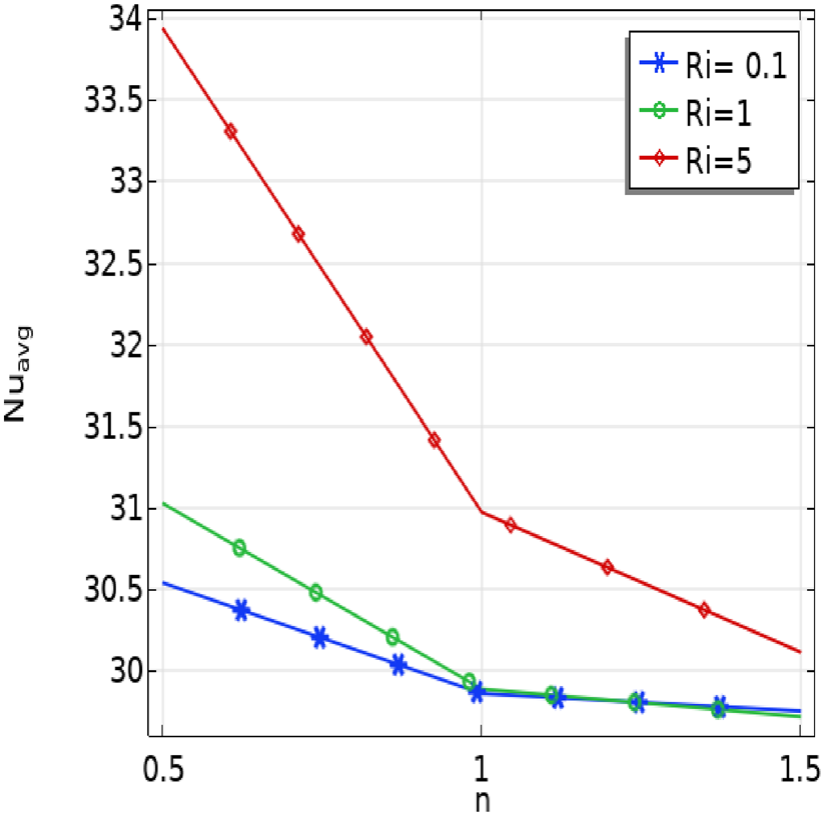
The effect of power law $(n)$ and Richardson number (Ri) on ${Nu}_{avg}$ number.

In Fig. 10, the influence of power law indices ($n$) on the average Nusselt number at the baffles across various Richardson number (Ri) scenarios is explored. Transitioning to different fluid types with increased viscosity reveals an inverse relationship—higher values of power law indices ($n$) correspond to a decrease in the Nusselt number. Additionally, a noteworthy trend emerges as the Richardson number increases; however, it is counteracted by a decrease in the Nusselt number with a rising power law index ($n$).

**Figure 11:**
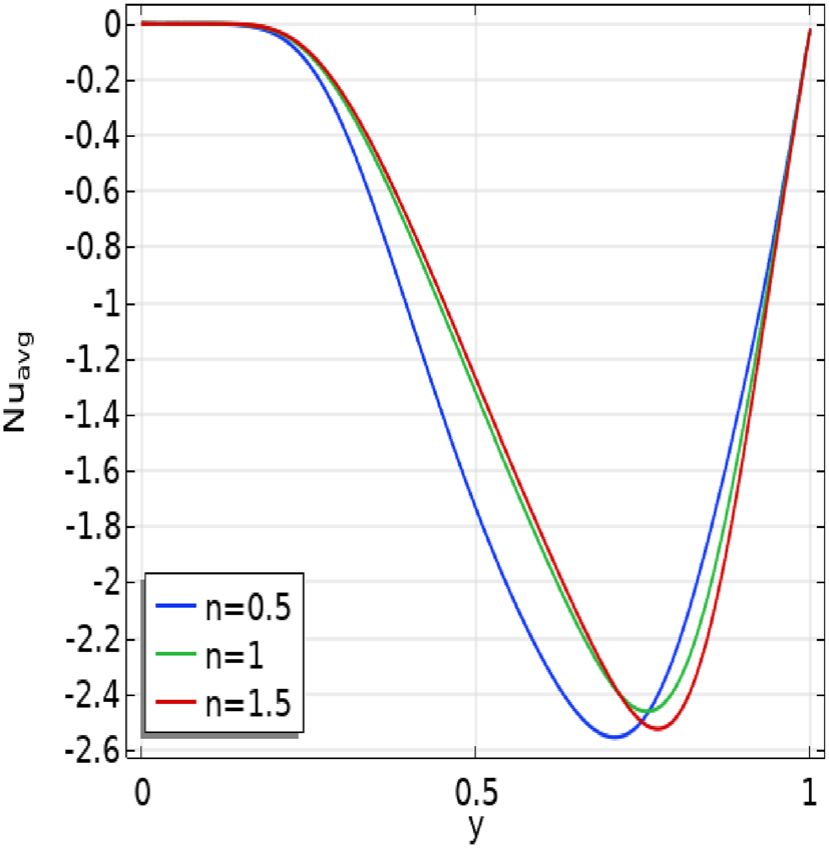
Graphs of average Nusselt number for various power law indices.

In Fig. 11 the graph of $({Nu}_{avg})$ for distinct values of power law indices at the baffle gap 0.6 is displayed. The curves exhibit a downward trend within the lower cavity for all values of n, followed by an upward trend. The shear thinning curve ($n < 1$) consistently remains below the shear thickening curve ($n > 1$) throughout the cavity. This implies that for the chosen parameter the shear thinning effect is weaker compared to the shear thickening effect.

## Conclusions

This investigation employed numerical simulations to investigate the thermal and fluid flow properties of a non-Newtonian fluid in a square ventilated enclosure under conditions of mixed convective flow. The enclosure was equipped with three distinct baffle gap sizes, namely (${b}_{g}$ = 0.2, 0.4, and 0.6). Furthermore, we assessed the impact of various parameters, including the power law index ($n$), Richardson number (Ri), Prandtl number (Pr), and Reynolds number (Re). The study also extends to the calculation average Nusselt number. The foremost findings can be summarized as follows:

1. The power law index ($n$) has a substantial impact on the convective heat transfer strength.
2. The rise in The Richards number from 0.1 to 5 enhanced the value of the average Nusselt number.
3. For varying Reynolds number levels, an increment in the Richardson number from 0.1 to 3 causes a proportional rise in the Nusselt number.
4. As the Prandtl number (Pr) transitions from 2 to 7, the Nusselt number experiences a corresponding increase across various Reynolds numbers.
5. The most favorable scenario occurs with a baffle gap of 0.6, wherein both the size and quantity of vortices increase with higher power law indices.
6. As the baffle gap widens, the zone dedicated to heat transfer expands, offering a larger canvas for the fluid within the cavity to absorb thermal energy.

## Data Availability

The datasets used and/or analysed during the current study are available from the corresponding author on reasonable request.

## List of symbols

$${C}_{p}$$: Specific heat ($${\text{J}}$$ (k$${{\text{g}})}^{-1}{{\text{K}}}^{-1}$$)
$${B}_{g}$$: Baffle gap
$Pr$: Prandtl number, dimensionless
$$p$$: Pressure $${({\text{Nm}}}^{-2}$$)
$$\overline{p }$$: Dimensionless pressure
$n$: Power law index, dimensionless
$$T$$: Temperature ($${\text{K}}$$)
$x,y$: Dimensional Cartesian coordinates $({\text{m}}$)
$\overline{X } \, \overline{Y }$: Dimensionless Cartesian coordinates
$Re$: Reynolds number, dimensionless
$$L$$: Cavity length $$({\text{m}})$$
$Ri$: Richardson number, dimensionless
$$u, v$$: Velocity components $$({\text{m}}$$ $${{\text{s}}}^{-1}$$)
$\overline{U } \, ,\overline{V }$: Dimensionless velocity components
$$m$$: Consistency parameter
$Gr$: Grashof number, dimensionless
$$g$$: Gravitational acceleration $$({\text{m}}$$ $${{\text{s}}}^{-2}$$)
$$k$$: Thermal conductivity (W $${{\text{m}}}^{-1}$$ $${{\text{K}}}^{-1}$$)
${Nu}_{avg}$: Average Nusselt number, dimensionless
${U}_{in}$: Velocity at the inlet port $({\text{m}}$ ${{\text{s}}}^{-1}$)

## Greek symbols

$$\rho $$: Fluid density $${\text{(kg}}\,{\text{m}}^{ - 3} )$$
$\theta$: Normalized temperature, dimensionless
$\beta$: Thermal expansion coefficient (${{\text{k}}}^{-1}$)
$$\mu $$: Dynamic viscosity (N s $${{\text{m}}}^{-2}$$)
τ: Shear stress
$$\eta $$: Apparent viscosity

## Supscript

$$C$$: Cold
$$H$$: Heated
$$in$$: Inlet port
$$out$$: Outlet port
$$w$$: Wall
$$avg$$: Average
